# Integrating iron metabolism-related gene signature to evaluate prognosis and immune infiltration in nasopharyngeal carcinoma

**DOI:** 10.1007/s12672-024-00969-3

**Published:** 2024-04-11

**Authors:** Jiaming Su, Guanlin Zhong, Weiling Qin, Lu Zhou, Jiemei Ye, Yinxing Ye, Chang Chen, Pan Liang, Weilin Zhao, Xue Xiao, Wensheng Wen, Wenqi Luo, Xiaoying Zhou, Zhe Zhang, Yonglin Cai, Cheng Li

**Affiliations:** 1 Department of Otolaryngology-Head and Neck Surgery, First Affiliated Hospital of Guangxi Medical University, Nanning, Guangxi China; 2 Department of Clinical Laboratory, Wuzhou Red Cross Hospital, #3-1 Xinxing Yi Road, Wuzhou, 543002 Guangxi China; 3 Guangxi Health Commission Key Laboratory of Molecular Epidemiology of Nasopharyngeal Carcinoma, Wuzhou Red Cross Hospital, Guangxi, China; 4 Department of Pathology, Wuzhou Red Cross Hospital, #3-1 Xinxing Yi Road, Wuzhou, 543002 Guangxi China; 5 Department of Pathology, Affiliated Tumor Hospital of Guangxi Medical University, Nanning, Guangxi China; 6 Key Laboratory of High-Incidence-Tumor Prevention & Treatment (Guangxi Medical University), Ministry of Education, Nanning, China

**Keywords:** Nasopharyngeal carcinoma, Iron metabolism, Prognostic signature, Tumor microenvironment, Ferroptosis

## Abstract

**Background:**

Dysregulation of iron metabolism has been shown to have significant implications for cancer development. We aimed to investigate the prognostic and immunological significance of iron metabolism-related genes (IMRGs) in nasopharyngeal carcinoma (NPC).

**Methods:**

Multiple Gene Expression Omnibus (GEO) and The Cancer Genome Atlas (TCGA) datasets were analyzed to identify key IMRGs associated with prognosis. Additionally, the immunological significance of IMRGs was explored.

**Results:**

A novel risk model was established using the LASSO regression algorithm, incorporating three genes (TFRC, SLC39A14, and ATP6V0D1).This model categorized patients into low and high-risk groups, and Kaplan–Meier analysis revealed significantly shorter progression-free survival for the high-risk group (*P* < 0.0001). The prognostic model’s accuracy was additionally confirmed by employing time-dependent Receiver Operating Characteristic (ROC) curves and conducting Decision Curve Analysis (DCA). High-risk patients were found to correlate with advanced clinical stages, specific tumor microenvironment subtypes, and distinct morphologies. ESTIMATE analysis demonstrated a significant inverse relationship between increased immune, stromal, and ESTIMATE scores and lowered risk score. Immune analysis indicated a negative correlation between high-risk score and the abundance of most tumor-infiltrating immune cells, including dendritic cells, CD8^+^ T cells, CD4^+^ T cells, and B cells. This correlation extended to immune checkpoint genes such as PDCD1, CTLA4, TIGIT, LAG3, and BTLA. The protein expression patterns of selected genes in clinical NPC samples were validated through immunohistochemistry.

**Conclusion:**

This study presents a prognostic model utilizing IMRGs in NPC, which could assist in assessing patient prognosis and provide insights into new therapeutic targets for NPC.

**Supplementary Information:**

The online version contains supplementary material available at 10.1007/s12672-024-00969-3.

## Introduction

Nasopharyngeal carcinoma (NPC) exhibits a unique geographic distribution, with a particularly high incidence in East and Southeast Asia [1]. Variations in dietary patterns, lifestyles, and exposure to adverse environmental conditions are probable key factors accounting for the differences in NPC incidence rates across diverse regions. Ethnicity-related genetic variations and the presence of the Epstein-Barr virus (EBV) are additional contributing factors [2]. However, the molecular pathogenesis of NPC is still not fully understood, and there are very few targeted therapeutic drugs developed for treating NPC.

Iron is an essential micronutrient that plays a critical role in multiple physiological processes, including oxygen transport, DNA synthesis and repair, and cellular metabolism [3]. In 2012, Dixond et al. reported an iron-related non-apoptotic cell death mode and named ferroptosis for the first time [4]. Since then, more and more evidence showed that ferroptosis is associated with a variety of diseases, including ischemic injury [5], neurodegenerative diseases [6, 7], autoimmune diseases [8], and inflammatory diseases [9]. Particularly, dysregulation of iron metabolism has been shown to have significant implications for the development of cancer [10]. When cells accumulate free iron, it leads to an increase in oxidative stress, reactive oxygen species (ROS) and DNA damage, ultimately promoting the occurrence and progression of cancer [11]. Abnormal iron metabolism has consistently been associated with cancer development and progression, making it a valuable tool for diagnosing and predicting outcomes. Dysregulation of key factors related to iron metabolism, such as lipocalin 2, transferrin receptor (TFR), and ferroportin (FPN), has been linked to unfavorable prognoses and decreased overall survival in different cancer types [12–14]. Additionally, iron homeostasis in tumors plays a role in modulating innate and adaptive immune responses, thereby influencing the tumor immune microenvironment [15]. Ferroptosis, a novel mode of regulated cell death, mainly relies on iron overload and abnormal accumulation of ROS [16]. Ferroptosis inducers have emerged as important players in anti-tumor effects. For example, erastin inhibits system xc-to reduce the synthesis of intracellular glutathione, resulting in the increase of ROS and subsequently inducing ferroptosis [4].

Iron metabolism is dysregulated in NPC, and changes in ferritin and lactotransferrin levels have been reported in NPC patients [17, 18]. Additionally, iron metabolism has been implicated in the occurrence of ferroptosis or resistance in NPC cells [19, 20]. NPC cells undergo cell death when exposed to ferroptosis inducers [21, 22]. Moreover, modulating specific iron metabolism related genes has been shown to sensitize NPC cells to radiotherapy [23–25]. However, the iron metabolism of NPC and the function of associated genes remain inadequately elucidated, and making a thorough investigation of its regulatory mechanism is of great significance for finding new therapeutic targets. In recent years, the pivotal role of gene sets in cancer prognosis has become increasingly prominence. Numerous studies have utilized bioinformatics methodologies to identify genes linked to crucial biological pathways [26–29], emphasizing the significance of gene signatures not only in cancer diagnosis but also in the development of robust prognostic models for cancer patients.

In this study, we applied high-throughput methods for a comprehensive analysis of identify iron metabolism-related genes, identify significant gene clusters linked to patient prognosis, developed diagnostic and prognostic models for NPC, explored their connection with immune infiltration, and validated the expression of selected targets. Our findings may enhance early NPC diagnosis and provide valuable insights for patient clinical outcomes.

## Materials and methods

### Data sources

The mRNA expression profiles were downloaded from the Gene Expression Omnibus (GEO) website (https://www.ncbi.nlm.nih.gov/geo/), including the GSE53819, GSE12452, GSE61218, and GSE102349 datasets, as shown in Table 1. The inclusion criterion for datasets was that the sample size in the control group was greater than five to reduce the sampling error and improve the reliability of differential analysis. Out of 113 patients in GSE102349, 88 had complete status data of progression-free survival (PFS). The clinical information in GSE102349 included the clinical stage, tumor morphology, tumor mutation burden (TMB), tumor microenvironment (TME)-based subtypes, the proportion of stromal tumor-infiltrating lymphocytes (TILs) and intratumoral TILs [30]. Subsequently, expression level of the EBV gene was evaluated based on genes that exhibited a significant correlation with EBV genes (Pearson coefficient > 0.3) through the single-sample Gene Set Enrichment Analysis (ssGSEA) algorithm. So far, no other public dataset with prognostic information for NPC patients has been found except GEO102349. We selected the head and neck squamous cell carcinoma (HNSCC) dataset with 499 tissue samples and complete clinical data, which were downloaded from The Cancer Genome Atlas (TCGA) database, as the validation cohort.

**Table 1:** Basic information of GEO datasets in this study

GEO accession	Platform	Experiment type	Sample
GSE53819	GPL6480	Expression profiling by array	36 (18 normal + 18 NPC)
GSE12452	GPL570	Expression profiling by array	41 (10 normal + 31 NPC)
GSE61218	GPL19061	Expression profiling by array	16 (6 normal + 10 NPC)
GSE102349	GPL11154	Expression profiling by high throughput sequencing	113 NPC

*GEO* Gene Expression Omnibus, *NPC* nasopharyngeal carcinoma

**Fig. 1:**
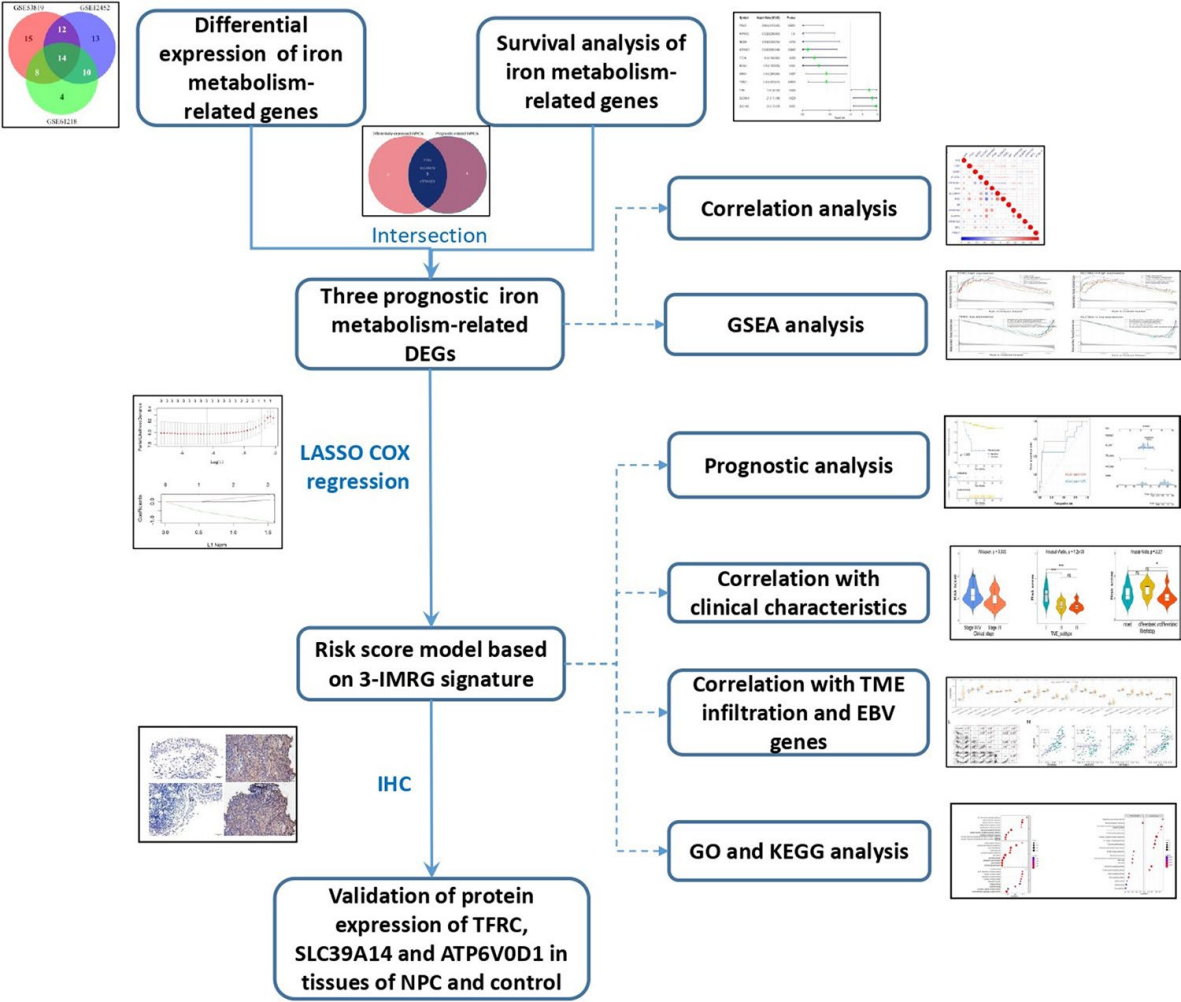
Study flow chart of this study. *DEG* differentially expressed gene, *IMRG* iron metabolism-related gene

A total of 66 IMRGs were obtained from the term of cellular iron ion homeostasis (GO: 0006879) on the AmiGo 2 website (http://amigo.geneontology.org/amigo). Additionally, 58 IMRGs were identified from the iron uptake and transport pathway (R-HSA-917937) on the Reactome website (https://reactome.org/). Subsequently, a total of 97 IMRGs were integrated for further analysis (see Additional file 1: Table S1). The flowchart for this study is depicted in Fig. 1.

### Analysis of differential expression and survival for iron metabolism-related genes

The R package “limma” [31] was utilized to analyze of IMRGs’ differential expression, and the p value was adjusted with “BH” method. Univariate Cox analysis was conducted to identify IMRGs with prognostic value. The key genes were identified by intersecting the IMRGs that exhibited differential expression with those IMRGs associated with prognosis.

### Construction of a prognostic model based on the key genes

An optimal prognostic risk model, utilizing the key genes, was built through the least absolute shrinkage and selection operator (LASSO) COX regression method. The optimal λ value was determined through 1000-fold cross-validation. Calculation of the risk score for each patient involved summing the product of the regression coefficient and the expression level of each key gene, expressed as follows: risk score = Σ (regression coefficient * gene expression level). Subsequently, the optimal cutoff value for the risk score, calculated with the “survminer” R package, was employed to classify NPC patients into high- and low-risk groups. To validate the prognostic model's accuracy, we performed time-dependent receiver operating characteristic (ROC) analysis and decision curve analysis (DCA). Additionally, a nomogram was established to estimate the prognostic model's probability. The accuracy of the risk score's predictions was further validated using the TCGA-HNSCC cohort.

### Analysis of immune microenvironment

The immune, stromal, and ESTIMATE scores of NPC patients were obtained by R package “ESTIMATE” [32]. To determine the proportion of immune-infiltrating cells in patients belonging to the high- and low-risk groups, ssGSEA was conducted by the R package “GSVA” [33]. The gene sets representing 24 [34] and 23 [35] types of immune cells used in this analysis were derived from existing literature.

### Functional enrichment analyses

Gene Ontology (GO) and Kyoto Encyclopedia of Genes and Genomes (KEGG) analyses of differentially expressed genes (DEGs) were conducted by the R package “clusterProfiler” [36] and “enrichplot”. To verify distinctions in biological functions and pathways, we conducted gene set enrichment analysis (GSEA). Next, we used the Search Tool for the Retrieval of Interacting Genes (STRING) (https://string-db.org) to predict protein functional interactions. For visualization of the protein–protein interaction (PPI) network and analysis of hub genes, Cytoscape software (version 3.9.1) [37] was utilized along with the cytoHubb [38] plugin with MCC algorithm.

### Immunohistochemistry staining assay

Paraffin-embedded tissue samples were collected from 47 individuals with NPC and 39 individuals with normal nasopharyngeal epithelium. This study was approved by the Ethics Committee of the Affiliated Tumor Hospital of Guangxi Medical University (No. LW2023146) and written informed content was obtained from the participants or the parents/legally authorized representatives of subjects who are under 16.

Tissue sections with a thickness of 3 µm were prepared from the tissue samples. Subsequently, tissue sections underwent deparaffinization using xylene and were then subjected to antigen retrieval. The sections were further treated to block endogenous peroxidase activity by incubating them with 3% hydrogen peroxide for one hour. Following this step, primary antibodies against TFRC (diluted 1:400, HPA028598, Sigma), SLC39A14 (diluted 1:400, ab106568, Abcam), and ATP6V0D1 (diluted 1:200, ab202897, Abcam) were applied, and the sections were left to incubate overnight at 4 °C. Afterward, the sections were incubated with the secondary antibody for 2 h at room temperature (PV-6000, ZSGB-BIO). Following the immunostaining, the sections were developed using 3,3ʹ-diaminobenzidine reagent (ZLI-9018, ZSGB-BIO) and counterstained with hematoxylin. Two experienced pathologists evaluated the immunostained tissue sections and scored the protein expression levels. Staining intensity was graded on a scale of 0 (absent), 1 (weak), 2 (moderate), or 3 (strong). The percentage of positive cells was scored as 0 (0%–10%), 1 (11%–50%), 2 (51%–80%), or 3 (81%–100%). The final immunoreactivity score was determined by multiplying the staining intensity score by the percentage score, resulting in a scale from 0 to 9.

### Statistical analysis

Statistical analysis and data visualization were carried out using R software (R Foundations for Statistical Computing version 4.2.1). To compare different groups, the Wilcoxon test and Kruskal–Wallis test were utilized, and Spearman correlation analysis was employed to investigate the relationship between gene expressions. Survival analyses, including Kaplan–Meier curves, log-rank tests, and COX regression analyses, were conducted using the “survival” package [39] and “survminer” packages. LASSO COX survival analysis was executed with “glmnet” package [40]. The “timeROC” package was utilized to generate time-dependent ROC curves and calculate the area under the curve (AUC). DCA was performed using the “ggDCA” package. The nomogram was created with the assistance of the “rms” and “regplot” packages. All p-values were calculated as two-sided, and statistical significance was established at a threshold of p < 0.05.

## Results

### Identification of differentially expressed IMRGs associated with prognosis in NPC

**Fig. 2:**
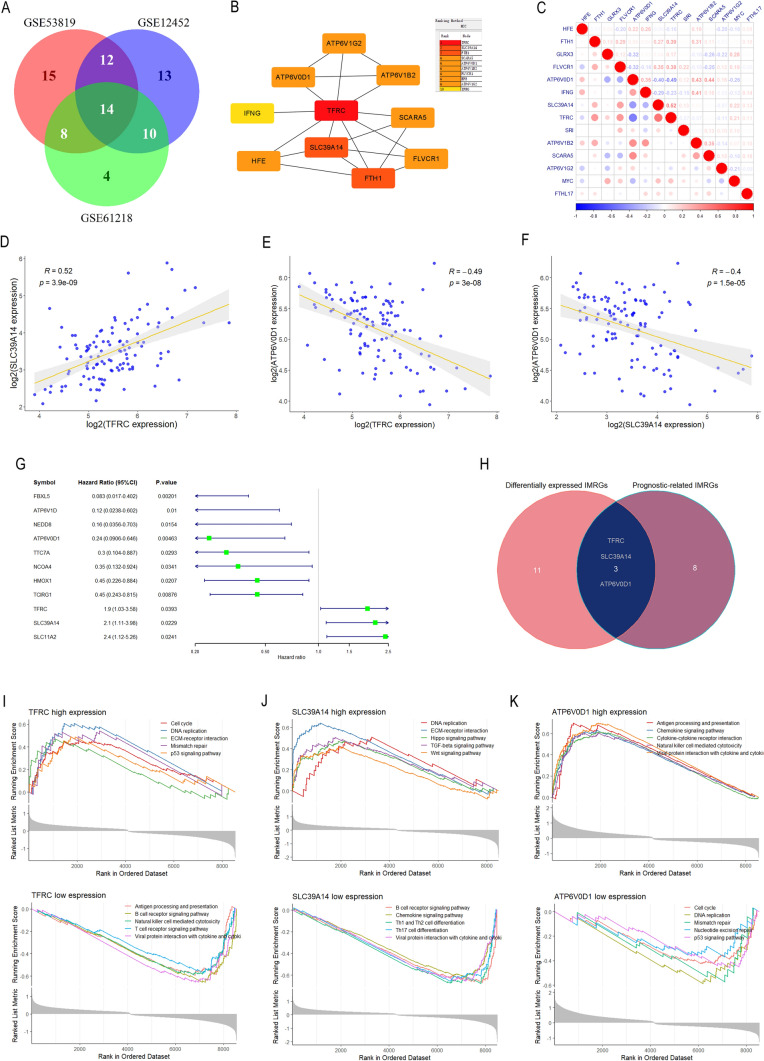
Identification of differentially expressed IMRGs associated with prognosis. **A** Venn diagrams analysis of differentially expressed IMRGs among GSE53819, GSE12452 and GSE61218. **B** Protein–protein interaction (PPI) network analysis of 14 differentially expressed IMRGs. **C** Correlation analysis of 14 differentially expressed IMRGs. **D** Correlations between TFRC and SLC39A14. **E** Correlations between TFRC and ATP6V0D1. **F** Correlations between SLC39A14 and ATP6V0D1. **G** Forest plot of the univariate Cox regression in 11 prognostic IMRGs in GSE102349. **H** Venn diagrams analysis of differentially expressed IMRGs and prognosis-related IMRGs. **I** GSEA of TFRC high- and low-expression. **J** GSEA of SLC39A14 high- and low-expression. **K** GSEA of ATP6V0D1 high- and low-expression. *GSEA* Gene set enrichment analysis, *IMRGs* iron metabolism-related genes

A total of 49, 49 and 36 differentially expressed IMGRs with p value < 0.05 were identified for GSE53819, GSE12452 and GSE61218, respectively. The intersection of the three datasets yielded 14 DEGs (Fig. 2A). PPI network analysis of these 14 DEGs revealed that the top 5 hub genes were TFRC, SLC39A14, FTH1, SCARA5, and ATP6V0D1 (Fig. 2B). The correlation plot of the 14 DEGs was presented in Fig. 2C, demonstrating significant strong correlations among the expression levels of TFRC, SLC39A14 and ATP6V0D1 (Fig. 2D–F).

We screened prognostic IMRGs in GSE102349 by using univariate COX regression analysis, and 11 IMRGs were found to be significantly associated with PFS in NPC patients (Fig. 2G). The intersection between 14 differential IMRGs and 11 IMRGs with prognostic value yielded 3 key genes (TFRC, SLC39A14 and ATP6V0D1) (Table 2, Fig. 2H).

**Table 2:** Overview of comprehensive features of key IMRGs

Gene	Chromosomal assignment	Protein localization	Biological function	Referenced
TFRC	3q29	Cytomembrane	Binding to transferrin and mediating iron uptake by cells through endocytosis	15109490
ATP6V0D1	16q22.1	Membrane, intracellular	Encoding a component of vacuolar ATPase (V-ATPase), a multisubunit enzyme that mediates acidification of eukaryotic intracellular organelles. In aerobic conditions, involved in intracellular iron homeostasis, thus triggering the activity of Fe(2 +) prolyl hydroxylase (PHD) enzymes	33065002 28296633
SLC39A14	8p21.3	Membrane, intracellular	Electroneutral transporter of the plasma membrane mediates the cellular uptake of the divalent metal cations zinc, manganese, and iron. Mediating the transport from endosomes to the cytosol of iron endocytosed by transferrin	15642354 29621230 20682781

GSEA was used to identify the signaling pathways that differ in NPC between high and low expressions of TFRC, SLC39A14 and ATP6V0D1. The enriched pathways in TFRC high expression phenotype included mismatch repair, ECM-receptor interaction, DNA replication, p53 signaling pathway, and cell cycle (Fig. 2I). The enriched pathways in SLC39A14 high expression phenotype included ECM-receptor interaction, DNA replication, TGF-β signaling, Hippo signaling, and Wnt signaling (Fig. 2J). The enriched pathways in ATP6V0D1 high expression phenotype included antigen processing and presentation, chemokine signaling, viral protein interaction with cytokine and cytokine receptor, cytokine-cytokine receptor interaction, and natural killer cell mediated cytotoxicity (Fig. 2K).

### Construction of a prognostic model based on the IMRGs signature

**Fig. 3:**
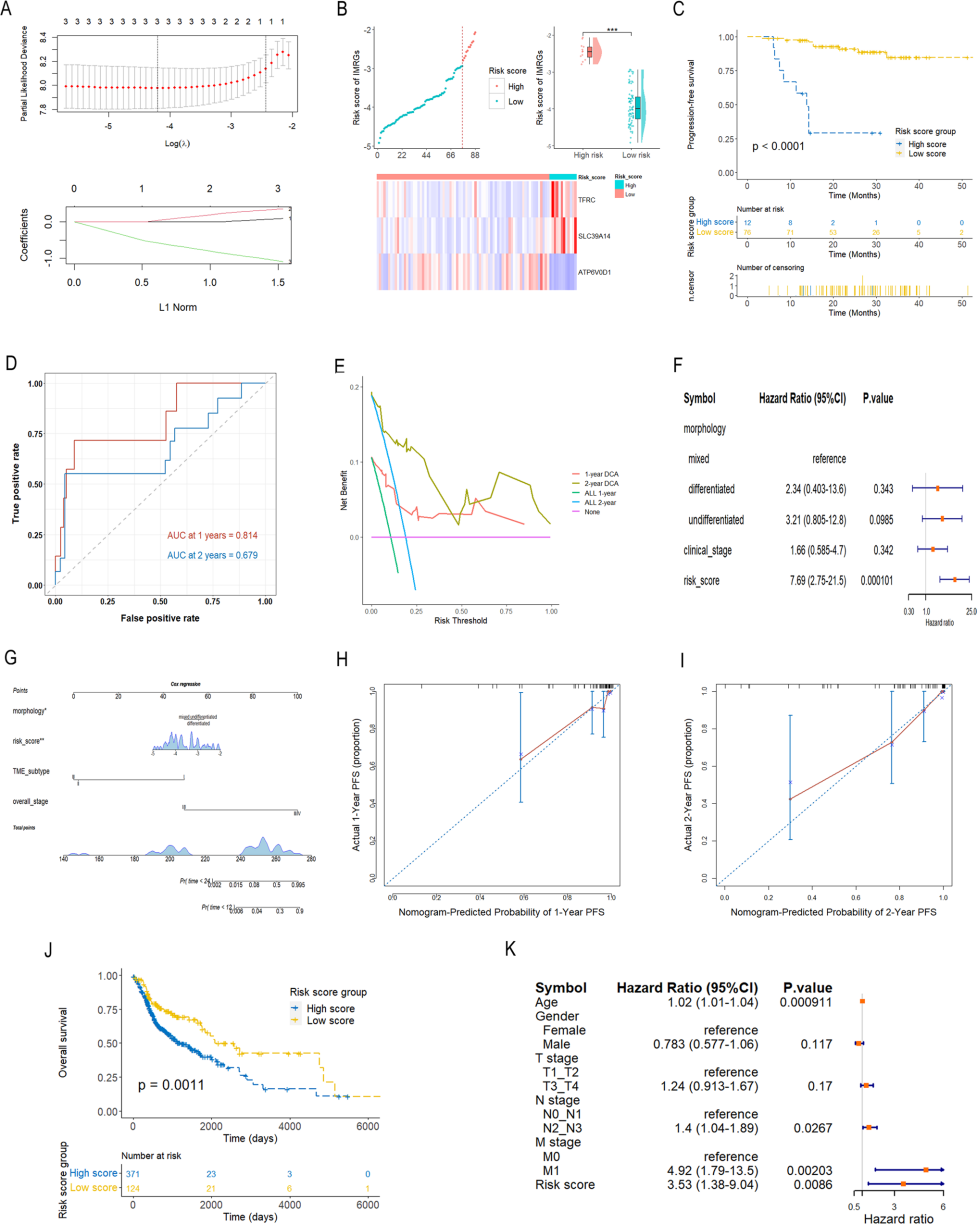
Prognostic model based on the 3-IMRG signature. **A** LASSO COX analysis was performed to construct a prognostic model using the 3-IMRG signature in GSE102349. **B** The survival status of NPC patients, distribution of risk score, and heatmap depicting the expression levels of the 3 IMRGs are displayed. **C** Kaplan–Meier survival curve illustrates the PFS of NPC patients categorized into low- and high-risk groups. **D** The prognostic value of the risk model for predicting 1- and 2-year PFS of NPC patients was evaluated using time-dependent ROC curves. **E** Decision curve analysis assesses the net clinical benefit of the risk model for predicting 1- and 2-year PFS of NPC patients. **F** Multivariate COX regression analysis demonstrates the prognostic significance of the risk score for NPC patients. **G** A nomogram is presented for predicting 1- and 2-year PFS of NPC patients. **H**, **I** Corresponding calibration curves are shown for evaluating the accuracy of 1- and 2-year PFS predictions for NPC patients. **J** Kaplan–Meier survival curve depicts the survival difference between high- and low-risk groups based on the 3-IMRG signature in the TCGA-HNSCC cohort. **K** Multivariate COX regression analysis assesses the prognostic value of the risk score in the TCGA-HNSCC cohort. *DP* disease progression, *HNSCC* head and neck squamous cell carcinoma, *IMRGs* iron metabolism-related genes, *OS* overall survival, *PF* progression-free, *PFS* progression-free survival. ***p < 0.001

LASSO COX regression analysis was applied to develop a prognostic model for NPC, utilizing the expression data from the 3 pivotal genes (TFRC, SLC39A14 and ATP6V0D1) (Fig. 3A). The risk score was calculated by the following: 0.06376 * expression level of TFRC + 0.3188 * expression level of SLC39A14 − 1.0073 * expression level of ATP6V0D1. NPC patients were categorized into high-risk and low-risk groups based on the optimal risk score cutoff value (Fig. 3B). A Kaplan–Meier analysis demonstrated that patients in the high-risk group experienced shorter PFS (Fig. 3C). Time-dependent ROC curve analysis indicated that the ROC AUC at 1-year and 2-year reached 0.814 (95%CI 0.634–0.992) and 0.679 (95%CI 0.494–0.864), respectively (Fig. 3D), indicating that risk score based on the 3-IMG signature had an excellent prognostic efficiency. Subsequently, the decision curve analysis demonstrated that the longer the survival time, the greater the net benefit of risk score model (AUDC = 0.033 and 0.078 at 1- and 2-year, respectively) (Fig. 3E). Multivariate Cox regression analysis revealed the potential of the risk score as an independent predictor for PFS in NPC (HR = 7.69, 95% CI 2.75–21.5) (Fig. 3F). Nomograms were created to predict 1-year and 2-year PFS, incorporating the risk score, morphology, TME subtype, and clinical stage (Fig. 3G). The C-index for the PFS nomogram was 0.890 (95%CI 0.817–0.962), affirming the model's reliability. Calibration curves results displayed consistency between the predicted and observed outcomes for the 1- and 2-year PFS, indicating that the nomogram could be predictive accuracy (Fig. 3H, I).

The predictive performance of the risk score based on the 3-IMG signature was validated using the TCGA-HNSCC cohort. The result of Kaplan–Meier analysis verified that HNSCC patients with high risk scores had poor overall survival (Fig. 3J). Moreover, COX regression model was used to build a multivariate prognostic model according to the age, gender, T, N, and M stages and risk score (Fig. 3K). The risk score, as well age, N, and M stages, were determined to be independent prognostic factors.

### Characteristics of the risk score based on IMRGs signature

**Fig. 4:**
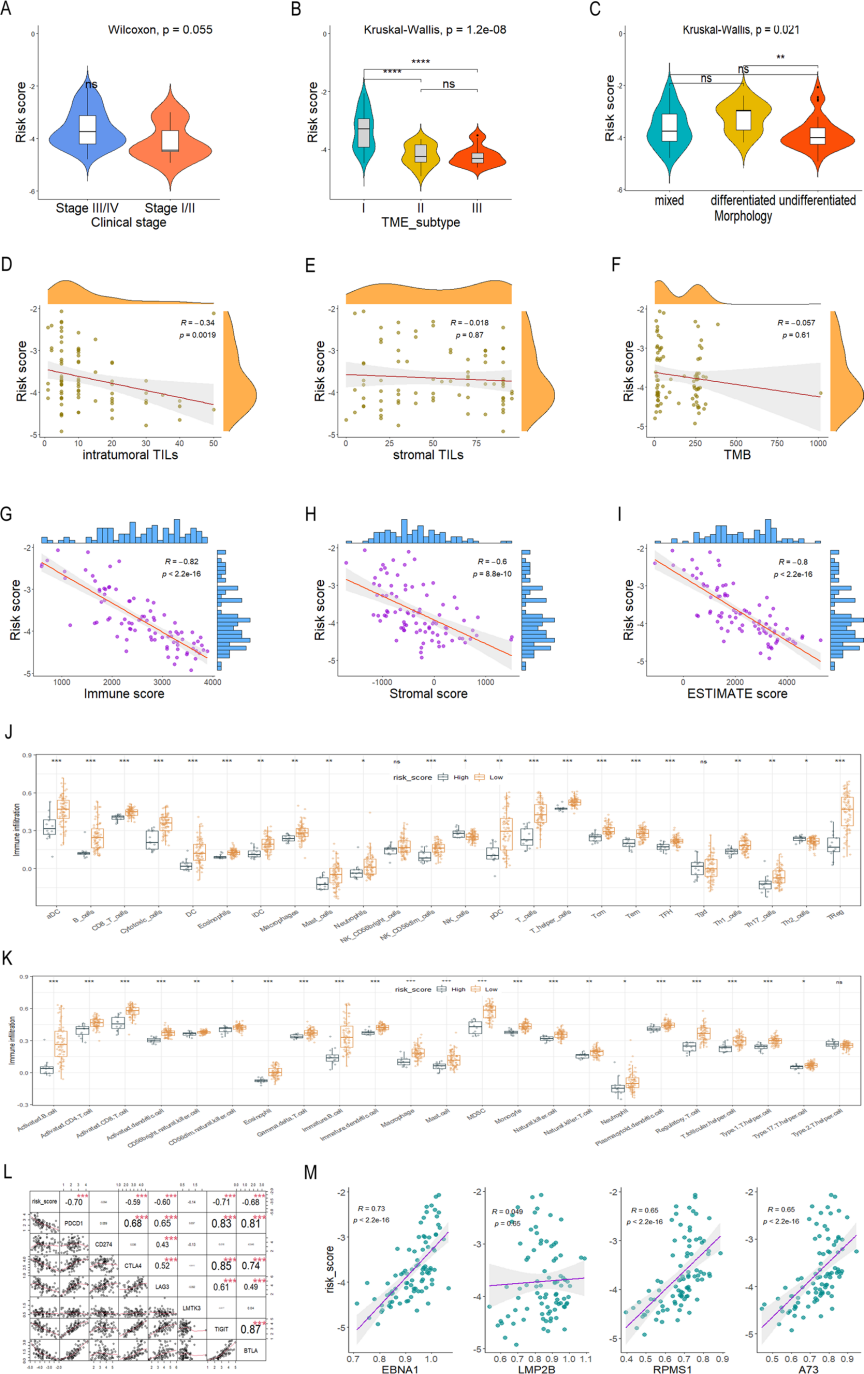
The relationship between the risk score and clinical features of NPC patients. The risk score is compared among different clinical stages (**A**), TME subtypes (**B**), and morphology (**C**). The correlation between the risk score and intratumoral TILs (**D**), stromal TILs (**E**), and TMB (**F**). Additionally, the correlation between the risk score and immune score (**G**), stromal score (**H**), and ESTIMATE score (**I**) is shown. Infiltration proportion of 24 (**J**) and 23 (**K**) types of immune cells is compared between high- and low-risk groups. Furthermore, the correlation between the risk score and the expression of immune checkpoint genes (**L**) and EBV genes (**M**) is examined. *TILs* tumor-infiltrating lymphocytes, *TME* tumor microenvironment, *TMB* tumor mutation burden. *p < 0.05, **p < 0.01, ***p < 0.001

Patients in early clinical stages (I–II) generally exhibited lower risk scores compared to those in advanced stages (III-IV), although the p-value did not reach statistical significance (Fig. 4A). The risk score was the highest in patients with TME subtype I than those with subtype II and III (Fig. 4B). The patients with undifferentiated morphology had lower risk score than those with differentiated morphology (Fig. 4C). The risk score exhibited a negatively association with the proportion of intratumoral TILs (Fig. 4D) and was not related to the proportion of stromal TILs and TMB (Fig. 4E, F). The above analysis results were consistent with the previous report [30]. Patients in TME subtype I had the worst prognosis. Subtype I is the only subtype that contains samples with differentiated morphology, with relatively few undifferentiated samples.

The ESTIMATE analysis highlighted a significant correlation between increased immune, stromal, and ESTIMATE scores and a decreased risk score (Fig. 4G–I). GSVA algorithm was performed to analyze the proportions of 24 and 23 kinds of immune-infiltrating cells in TME (Fig. 4J, K). Our results reveal that the low-risk group exhibits higher levels of most immune cell populations. Taken together, our results suggested a close association between the risk score, which is based on the 3-IMRG signature, and tumor immunity and might affect the response of NPC patients to immunotherapy. The risk score demonstrated a negative correlation with the expression of immune checkpoint genes, including PDCD1 (PD-1), CTLA4, LAG3, TIGIT, and BTLA (Fig. 4L). Additionally, it exhibited a positive correlation with the expression of EBV genes (Fig. 4M).

### Functional enrichment analysis in high- and low-risk groups

**Fig. 5:**
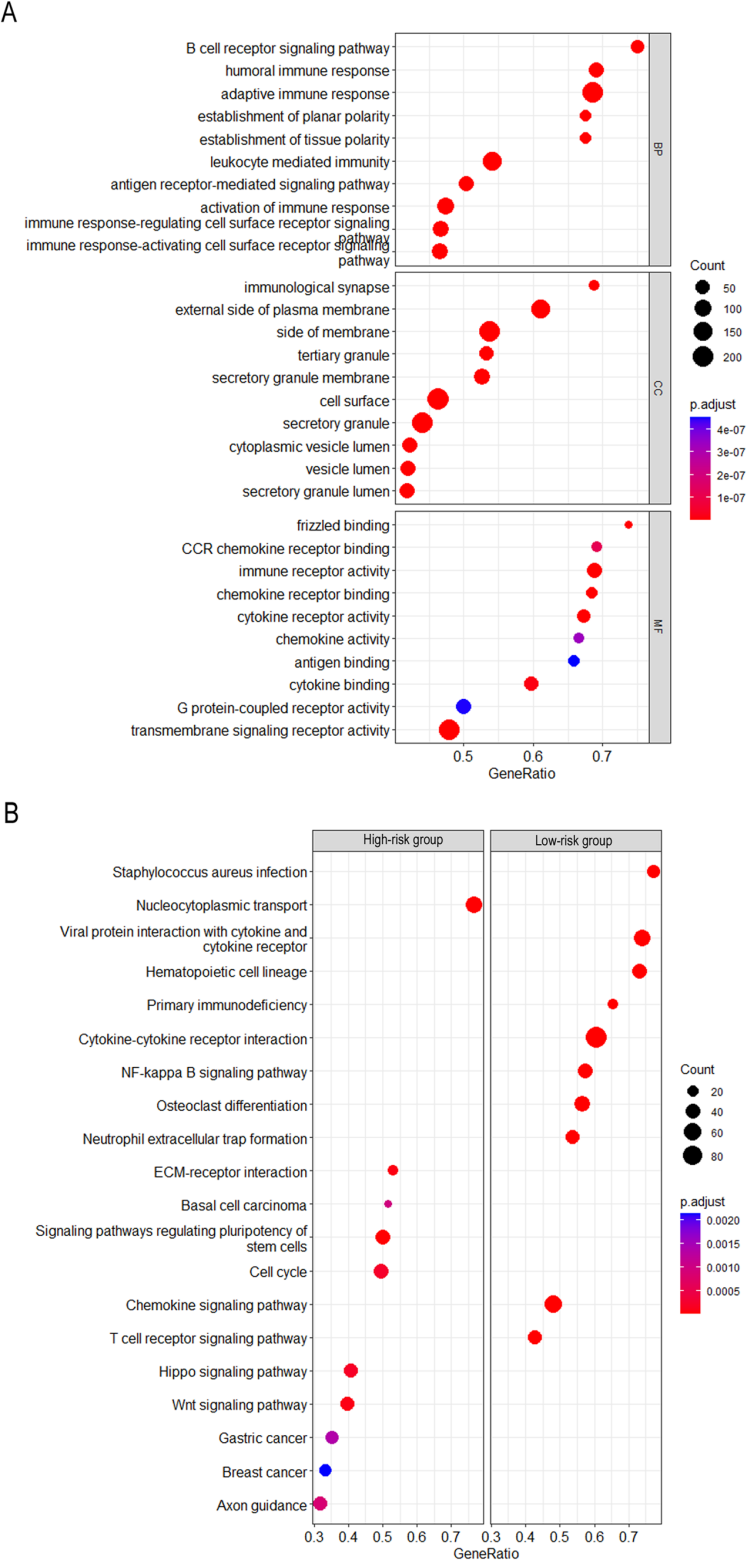
Gene enrichment analysis of in high- and low-risk groups. **A** Gene ontology (GO) enrichment analysis. **B** Kyoto Encyclopedia of Genes and Genomes (KEGG) pathway analysis. *BP* biological process, *CC* cellular component, *MF* molecular function

GO and KEGG enrichment analyses were performed to explore the biological functions of the risk score based on 3-IMRG signature. During GO analysis (Fig. 5A), significantly enriched GO cellular component (CC) included external side of plasma membrane, side of membrane, immunological synapse tertiary granule, and secretory granule membrane. Significantly enriched GO terms associated with the molecular function (MF) included frizzled binding, CCR chemokine receptor binding, immune receptor activity, chemokine receptor binding, and cytokine receptor activity. In addition, significantly enriched GO terms related to biological process (BP) included humoral immune response, adaptive immune response, B cell receptor signaling pathway, establishment of planar polarity, and establishment of tissue polarity. On the other hand, the top five KEGG pathways related to the high-risk group included nucleocytoplasmic transport, ECM-receptor interaction, cell cycle, signaling pathways regulating pluripotency of stem cells, and Hippo signaling pathway, and the KEGG pathways related to the low-risk group included cytokine-cytokine receptor interaction, chemokine signaling pathway, NF-κB signaling pathway, viral protein interaction with cytokine and cytokine receptor, and T cell receptor signaling pathway (Fig. 5B). The above results suggested that the signaling pathways and biological behaviors enriched by iron metabolism-related DEGs were related to TME or immune functions.

### Verification of protein expression of TFRC, SLC39A14 and ATP6V0D1 in clinical samples by using immunohistochemistry

**Fig. 6:**
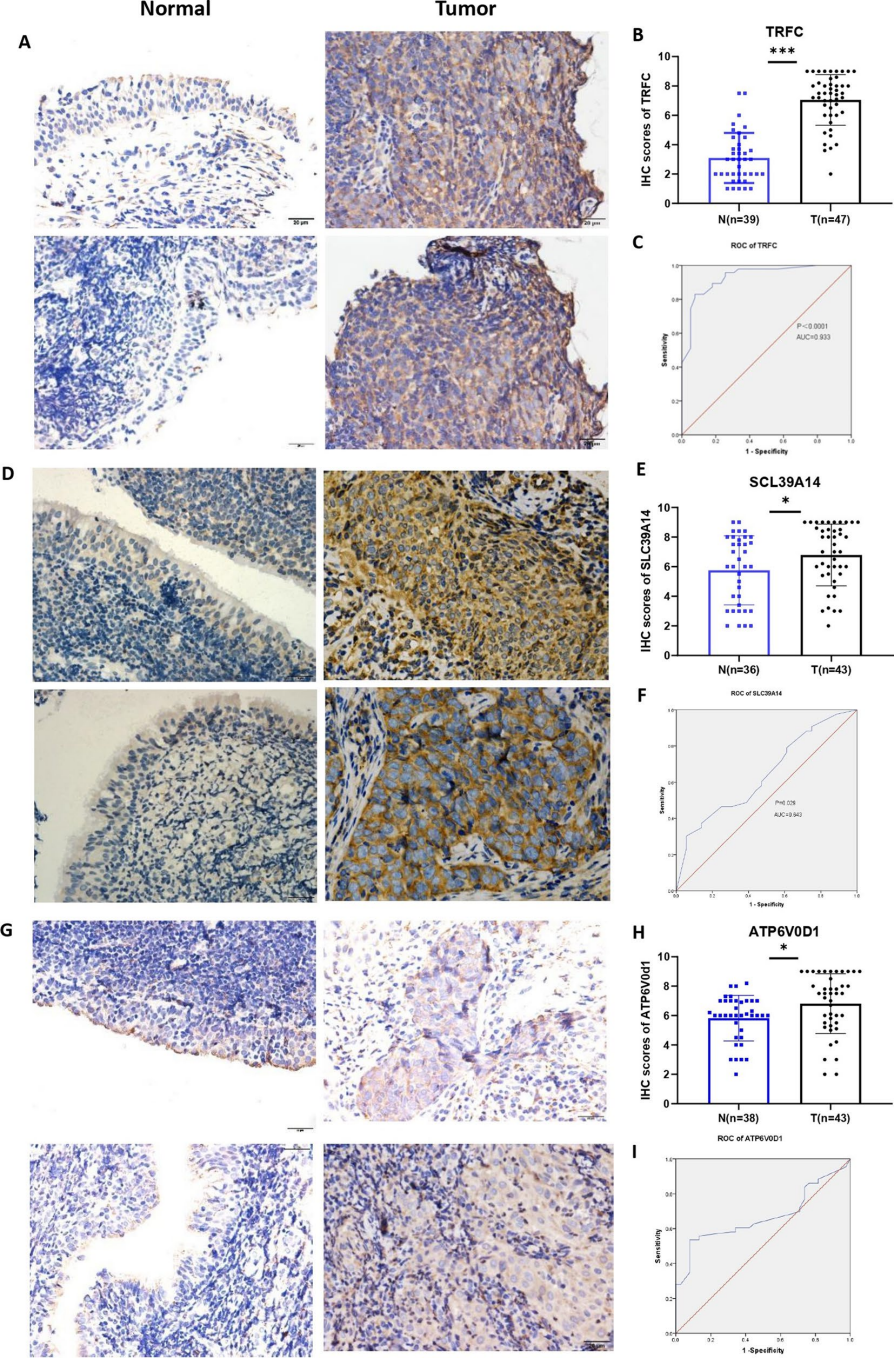
Immunohistochemistry of TFRC, SLC39A14, and ATP6V0D1. **A**, **B** TFRC protein levels in NPC tissue expression. **C** Diagnostic efficacy of TFRC in NPC based on protein level. **D**, **E** SLC39A14 protein levels in NPC tissue expression. **F** Diagnostic efficacy of SLC39A14 in NPC based on protein level. **G**, **H** ATP6V0D1 protein levels in NPC tissue expression. **I** Diagnostic efficacy of ATP6V0D1 in NPC based on protein level. *AUC* Area under curve. *p < 0.05, ***p < 0.001

The protein expression levels of TFRC and SLC39A14 were significantly elevated in NPC tissues than those in normal nasopharyngeal epithelium tissues (Fig. 6A, B, D, E), consistent with previous differential analysis of transcriptome. Nonetheless, ATP6V0D1 expression in NPC tissues exceeded that in normal nasopharyngeal epithelial tissues (Fig. 6G, H), contrary to the result of transcriptome analysis. Subsequently, ROC curve analysis was performed on the immunohistochemistry-derived staining scores for TFRC, SLC39A14, and ATP6V0D1 (Fig. 6C, F, I), further confirming the potential of these proteins in diagnosing NPC.

## Discussion

Several studies have investigated the characteristics of iron metabolism signatures in cancers. For example, the prognostic relevance of IMRGs in papillary thyroid carcinoma (PTC) was analyzed using data from the TCGA and GEO database. The expressions of Sideroflexin 3 (SFXN3) and TFR2 were elevated in PTC tissues, a knocked down of these two genes, respectively, has been demonstrated to inhibit the proliferation of PTC cells [41]. For lung adenocarcinoma, the level of STEAP1 and STEAP2, which have the ability to reduce Fe(3+) to Fe(2+) and stimulate the cellular iron uptake, were negatively related to prognosis of patients based on multiple databases [42]. Copper, like iron, is an essential cofactor for all organisms. Tsvetkov et al. firstly found that copper ionophore could induce a distinctive form of cell death, namely as cuproptosis [43]. Selenium and zinc metabolism have also been documented to play significant roles in the development, progression, and therapeutic responses in various cancers [44, 45].

Genomic approaches using relevant databases to identify key cancer pathways can facilitate the search for novel and critical biomarkers and aid in disease diagnosis and treatment [46]. In this study, we performed a systematic analysis of the dysregulation of IMRGs in NPC using the GEO transcriptome and clinical data. Our analysis revealed that 14 genes were dysregulated in NPC, and further PPI network analysis identified TFRC (also known as TFR1), SLC39A14, FTH1, SCARA5, and ATP6V0D1 as the top hub genes. We then utilized the LASSO COX regression model to establish a risk score using three genes associated with iron metabolism, including TFRC, SLC39A14, and ATP6V0D1. Using this risk score, we constructed a nomogram for predicting the PFS of NPC. A higher risk score, determined by the 3-IMRG signature was associated with worse PFS in NPC. Additionally, similar finding was observed in TCGA-HNSCC cohort. Moreover, differential expression analysis of the data from GEO demonstrated that TFRC and SLC39A14 were highly expressed in NPC tissues, which was further confirmed by IHC experiments using clinical tissue samples. However, the protein expression of ATP6V0D1 contradicted the findings from the bioinformatics analysis of the GEO dataset. This discrepancy could potentially be attributed to transcriptional or post-transcriptional modifications of ATP6V0D1.

There are conflicting findings regarding the expression patterns of SLC39A14 and its effects on different forms of cancer in humans. In renal cell carcinoma (RCC) tissues, like in NPC, the mRNA expression of SLC39A14 was significantly enhanced; inhibiting SLC39A14 might impair the migration, invasion, proliferation, and epithelial-mesenchymal transition (EMT) of RCC cells [47]. However, in prostate cancer patients, reduced expression of SLC39A14, which serves as a tumor suppressor, may lead to malignant phenotypes and aggressive tumor progression [48]. On the other hand, higher SLC39A14 expression in gastric cancer patients was closely associated with a favorable overall survival [49]. SLC39A14 transferred non-transferrin-bound iron into the mouse liver resulting in ferroptosis to promote liver fibrosis [50]. In addition, SLC39A14 was involved in the activation of pulmonary macrophages, and inhibition of SLC39A14 significantly enhanced the expression levels of inflammatory factors IL-6 and TNFα [51, 52]. These indicate the critical role of SLC39A14 in ferroptosis and regulation of tumor immune microenvironment.

ATP6V0D1 encodes a component of vacuolar ATPase (V-ATPase), which plays a role in maintaining intracellular iron homeostasis in aerobic conditions. It activates the Fe(2+) prolyl hydroxylase (PHD) enzymes, initiating the hydroxylation of HIF1A and its subsequent degradation via the proteasome [53]. Inhibition of V-ATPase disrupts intracellular iron levels and hinders the uptake of transferrin [54, 55], Affirming V-ATPase's role in regulating iron through the process of clathrin-mediated endocytosis [54]. The involvement of V-ATPase extends beyond iron acquisition and its conversion to Fe(2+); it is also essential for liberating iron from ferritin reserves through ferritinophagy [53]. The naturally occurring V-ATPase inhibitor archazolid disrupts the recycling of endocytosed TFR, resulting in cytosolic iron depletion in breast cancer cells, ultimately inducing cell death [56].

TFRC encodes transferrin receptor protein 1, which is closely related to ferroptosis, and plays an important role in iron import from extracellular environment to cells [57]. The increased demand for iron uptake in a variety of solid cancers leads to high expression of TFRC, such as esophageal squamous cell carcinoma, lung cancer, breast cancer and renal cell carcinoma, it is associated with unfavorable prognosis [58]. TFRC participates in the formation of the T cell immune synapse and influences T cell receptor function [59]. In addition, TFRC was associated with the expression of multiple immune-related target genes [60]. The expression of TFRC is significantly upregulated in breast cancer and pancreatic cancer, and TFRC is correlated with the infiltrating abundance and immune response of some immune cells [61, 62]. Consistent with our study, TFRC links iron metabolism and immunity, which together influence the tumor immune microenvironment. The m6A modification is also involved in the regulation of the immune microenvironment in inflammation [63]. Insulin-like growth factor 2 mRNA-binding protein 2 (IGF2BP2) can preserve the stability of TFRC mRNA through m6A modifications, thereby increasing TFRC protein levels in colorectal cancer (CRC) cells and facilitating the growth of CRC [64]. Targeting IGF2BP2 and TFRC may represent innovative approaches for the diagnosis and treatment of cancer.

Increased redox-active labile iron pool promotes cancer growth and metastasis, but increased iron-induced cytotoxic lipid radicals also cause ferroptosis [65]. Studies have shown that iron overload can increase the risk of hepatocellular carcinoma (HCC) and reduce the survival rate of patients [66]. In tumors with iron overload, the key signaling pathway JAK/STAT regulates FPN and hepcidin to promote tumor progression [67]. In this context, novel pharmacological agents have emerged as potential candidates for orchestrating iron metabolism and influencing the JAK/STAT signaling cascade within HCC. The intricate interplay between iron homeostasis and signaling pathways underscores the complexity of cancer biology and the multifaceted potential for therapeutic intervention. In the future, cancer therapies against tumorigenic cancer stem cells may target iron homeostasis, such as small-molecule 5-ALA-PDT, ferroptosis, and inhibitors [65].

The immune analysis results revealed a pronounced negative correlation between the risk score and TILs proportion within the tumor. In the low-risk group, the abundance of most immune cells increased. Iron homeostasis appears to be particularly crucial for immune cells exerting anti-tumor functions. Transferrin receptor, a crucial protein in early T cell differentiation, is indispensable. Decreased transferrin and its receptor may lead to reduced thymic cell numbers [68, 69]. Imbalanced iron homeostasis has also been shown to affect the ratio of CD4^+^ T cells to CD8^+^ T cells. For example, patients with iron overload due to β-thalassemia exhibit increased CD8^+^ T cells and decreased CD4^+^ T cells [70]. It has been demonstrated that interferon gamma released by CD8^+^ T cells inhibits system xc-glutamate-cystine antiporter, limiting the cystine uptake by tumor cells, thereby enhancing tumor cell lipid peroxidation and inducing iron-dependent cell death. This effect can be activated by PD-1 blockade immunotherapy, enhancing the sensitivity of tumor cells to iron death induced by lipid peroxidation [71]. Tumor-associated macrophages (TAMs) have a pivotal role in tumor immunity within the tumor microenvironment. TAMs can be divided into M1-type macrophages with pro-inflammatory phenotypes and M2-type macrophages with anti-inflammatory phenotypes [72]. M1 macrophages exhibit low levels of iron transport proteins and high levels of ferritin, indicative of an iron-chelating phenotype, whereas M2 macrophages show high levels of iron transport proteins and low levels of ferritin, representing an iron-supply phenotype [15]. M1 macrophages induce reactive ROS through the NF-kB pathway-mediated nicotinamide adenine dinucleotide phosphate oxidase (NOX), thus participating in the induction of iron death. Promoting the transition of M2 macrophages to M1 macrophages is a novel strategy to enhance tumor immunity. For example, the reprogramming of macrophages using hyaluronic acid-modified superparamagnetic iron oxide nanoparticles (HIONs), more effectively generating ROS and bioactive factors (TNFα and IL-6), inhibiting tumor growth, and inducing the transition of M2 macrophages to M1 macrophages through paracrine mechanisms [73]. Additionally, iron overload-induced high levels of ROS enhance the activity of p300/CBP acetyltransferase, leading to increased p53 acetylation and promoting the shift of macrophages towards the M1 subtype [74]. Therefore, intervening in the iron death pathway holds promise to induce iron-related toxicity in tumor cells, further strengthening the tumor immune response, and providing new strategies and possibilities for immunotherapy. Furthermore, we also reported that the risk score displayed a pronounced negative correlation with the expression of immune checkpoint genes including PDCD1 (PD-1), LAG3, CTLA4, TIGIT, and BTLA. IMRGs could potentially forecast or impact the efficacy of immune therapy in NPC patients.

However, our study does have certain limitations. The data we used are sourced from the GEO database, which means they might be influenced by experimental conditions, sample characteristics, and potential confounding factors. Therefore, while our research highlights the potential predictive significance of iron metabolism-related genes in NPC, further validation through prospective studies is necessary to ensure consistent and reliable outcomes.

## Conclusion

In summary, we have identified key genes involved in iron metabolism in NPC, including TFRC, ATP6V0D1 and SLC39A14. Additionally, we have developed a diagnostic and prognostic model for NPC, which holds promise as a potential new therapeutic approach for NPC patients.

### Supplementary Information

Additional file1 (DOCX 20 KB)

## Data Availability

The relevant data of NPC patients used in this study were obtained from GEO and TCGA databases.
