# Supplementary material for: Integrating iron metabolism-related gene signature to evaluate prognosis and immune infiltration in nasopharyngeal carcinoma
Source: Discov Oncol. 2024 Apr 11;15:112. doi: 10.1007/s12672-024-00969-3 (PMC11009181; doi:10.1007/s12672-024-00969-3)
Supplement: Supplementary file 1 — Additional file1 (DOCX 20 KB) [file 12672_2024_969_MOESM1_ESM.docx]

**Supplementary Table 1. Iron metabolism-related genes**

|  | Gene symbol |
| --- | --- |
| 1 | ABCB6 |
| 2 | ABCB7 |
| 3 | ABCG2 |
| 4 | ACO1 |
| 5 | ALAS2 |
| 6 | ATP13A2 |
| 7 | ATP6AP1 |
| 8 | ATP6V0A1 |
| 9 | ATP6V0A2 |
| 10 | ATP6V0A4 |
| 11 | ATP6V0B |
| 12 | ATP6V0C |
| 13 | ATP6V0D1 |
| 14 | ATP6V0D2 |
| 15 | ATP6V0E1 |
| 16 | ATP6V0E2 |
| 17 | ATP6V1A |
| 18 | ATP6V1B1 |
| 19 | ATP6V1B2 |
| 20 | ATP6V1C1 |
| 21 | ATP6V1C2 |
| 22 | ATP6V1D |
| 23 | ATP6V1E1 |
| 24 | ATP6V1E2 |
| 25 | ATP6V1F |
| 26 | ATP6V1G1 |
| 27 | ATP6V1G2 |
| 28 | ATP6V1G3 |
| 29 | ATP6V1H |
| 30 | BMP6 |
| 31 | BOLA2 |
| 32 | BOLA2B |
| 33 | CAND1 |
| 34 | CCDC115 |
| 35 | CP |
| 36 | CUL1 |
| 37 | CYB561 |
| 38 | CYB561A3 |
| 39 | CYBRD1 |
| 40 | ERFE |
| 41 | FBXL5 |
| 42 | FLVCR1 |
| 43 | FRRS1 |
| 44 | FTH1 |
| 45 | FTH1P19 |
| 46 | FTHL17 |
| 47 | FTL |
| 48 | FTMT |
| 49 | FXN |
| 50 | GDF2 |
| 51 | GLRX3 |
| 52 | HAMP |
| 53 | HEPH |
| 54 | HEPHL1 |
| 55 | HFE |
| 56 | HIF1A |
| 57 | HJV |
| 58 | HMOX1 |
| 59 | HMOX2 |
| 60 | HPX |
| 61 | IFNG |
| 62 | IREB2 |
| 63 | ISCU |
| 64 | LCN2 |
| 65 | MCOLN1 |
| 66 | MYC |
| 67 | NCOA4 |
| 68 | NDFIP1 |
| 69 | NEDD8 |
| 70 | NUBP1 |
| 71 | RPS27A |
| 72 | SCARA5 |
| 73 | SKP1 |
| 74 | SLC11A1 |
| 75 | SLC11A2 |
| 76 | SLC22A17 |
| 77 | SLC39A14 |
| 78 | SLC39A8 |
| 79 | SLC40A1 |
| 80 | SLC46A1 |
| 81 | SLC6A9 |
| 82 | SMAD4 |
| 83 | SOD1 |
| 84 | SRI |
| 85 | STEAP2 |
| 86 | STEAP3 |
| 87 | STEAP4 |
| 88 | TCIRG1 |
| 89 | TF |
| 90 | TFR2 |
| 91 | TFRC |
| 92 | TMEM199 |
| 93 | TMPRSS6 |
| 94 | TTC7A |
| 95 | UBA52 |
| 96 | UBB |
| 97 | UBC |
